# Visible CCD Camera-Guided Photoacoustic Imaging System for Precise Navigation during Functional Rat Brain Imaging

**DOI:** 10.3390/bios13010107

**Published:** 2023-01-06

**Authors:** Yuhling Wang, Yu-Lin Chen, Chih-Mao Huang, Li-Tzong Chen, Lun-De Liao

**Affiliations:** 1 Institute of Biomedical Engineering and Nanomedicine, National Health Research Institutes, No.35, Keyan Road, Zhunan Town, Miaoli County 350, Taiwan; 2 Department of Biological Science and Technology, National Yang Ming Chiao Tung University, No.75 Po-Ai St., Hsinchu 300, Taiwan; 3 Department of Internal Medicine, Kaohsiung Medical University Hospital and Center for Cancer Research, Kaohsiung Medical University, No.100, Tzyou 1st Road, Sanmin Dist., Kaohsiung City 80756, Taiwan; 4 National Institute of Cancer Research, National Health Research Institutes, No.35, Keyan Road, Zhunan Town, Miaoli County 350, Taiwan

**Keywords:** photoacoustic (PA) imaging system, charged-coupled device (CCD) camera, photothrombotic ischemia (PTI), primary somatosensory cortex of the forelimb (S1FL), cerebral blood volume (CBV), oxygen saturation (SO_2_), cortical spreading depression (CSD)

## Abstract

In photoacoustic (PA) imaging, tissue absorbs specific wavelengths of light. The absorbed energy results in thermal expansion that generates ultrasound waves that are reconstructed into images. Existing commercial PA imaging systems for preclinical brain imaging are limited by imprecise positioning capabilities and inflexible user interfaces. We introduce a new visible charge-coupled device (CCD) camera-guided photoacoustic imaging (ViCPAI) system that integrates an ultrasound (US) transducer and a data acquisition platform with a CCD camera for positioning. The CCD camera accurately positions the US probe at the measurement location. The programmable MATLAB-based platform has an intuitive user interface. In vitro carbon fiber and in vivo animal experiments were performed to investigate the precise positioning and imaging capabilities of the ViCPAI system. We demonstrated real-time capturing of bilateral cerebral hemodynamic changes during (1) forelimb electrical stimulation under normal conditions, (2) forelimb stimulation after right brain focal photothrombotic ischemia (PTI) stroke, and (3) progression of KCl-induced cortical spreading depression (CSD). The ViCPAI system accurately located target areas and achieved reproducible positioning, which is crucial in animal and clinical experiments. In animal experiments, the ViCPAI system was used to investigate bilateral cerebral cortex responses to left forelimb electrical stimulation before and after stroke, showing that the CBV and SO_2_ in the right primary somatosensory cortex of the forelimb (S1FL) region were significantly changed by left forelimb electrical stimulation before stroke. No CBV or SO_2_ changes were observed in the bilateral cortex in the S1FL area in response to left forelimb electrical stimulation after stroke. While monitoring CSD progression, the ViCPAI system accurately locates the S1FL area and returns to the same position after the probe moves, demonstrating reproducible positioning and reducing positioning errors. The ViCPAI system utilizes the real-time precise positioning capability of CCD cameras to overcome various challenges in preclinical and clinical studies.

## 1. Introduction

A recent World Health Organization report noted that ischemic stroke is one of the top three causes of death worldwide [1]. Approximately 5.5 million people died of ischemic stroke in 2016, and approximately 116.4 million people suffered permanent disability after a stroke event [2]. These mortalities and disabilities have substantial emotional and economic impacts on families and society. Therefore, tools for understanding stroke or related cerebral diseases are indispensable for improving preclinical brain research. Commonly used stroke imaging systems include computed tomography (CT), magnetic resonance imaging (MRI), and positron emission tomography (PET) [3]. CT imaging systems use X-rays to penetrate the human body to obtain image signals, which are processed by a computer to obtain a three-dimensional image that can be postprocessed by software to form image information for various sections [4]. MRI scans employ powerful magnetic fields and radio waves to excite hydrogen atoms in the human body and generate detailed images of the interior of the human body, which are then processed by computers to obtain three-dimensional images and postprocessed by software to determine image information in various sections [5]. PET systems use isotope drugs (positron agents) that emit positrons and generate positrons by decay, which then collide with negatively charged electrons in tissues, resulting in positron annihilation events that release energy [3]. The detector identifies the energy released in both directions to form images [4].

According to previous reports, PET and MRI are commonly used in animal stroke models to observe the neuroprotective effects of drugs or electrical stimulation [6,7,8]. Existing diagnostic imaging instruments, such as CT and MRI, have accurate positioning capabilities, which can improve image positioning [9,10]. Since PET has no positioning capability, PET systems can be combined with medical imaging instruments such as CT or MRI to assist in positioning. For instance, PET-CT and PET-MRI systems are helpful for presenting and investigating images [11]. Precise positioning is an indispensable technology in both human and animal imaging research [12]. In summary, CT, MRI, PET-CT, and PET-MRI have localization capabilities and can be applied in lesion diagnoses, and photoacoustic systems must have accurate localization capabilities.

In recent years, photoacoustic (PA) techniques have been widely used in both preclinical and clinical settings [13,14,15], including tumor imaging [16,17], dermatology imaging [18], vascular imaging, musculoskeletal imaging, gastrointestinal imaging [19], and fat tissue imaging [20]. These PA imaging systems have emerged as powerful scientific research tools. PA imaging combines great spatial optical resolution with high acoustic penetration to develop multifunctional hybrid imaging systems that utilize optical absorption to produce high-contrast tissue images. Therefore, ultrasound (US)/PA imaging technology can provide researchers with functional and structural information about angiogenesis, hemoglobin oxygen saturation [8,13,21,22], and total hemoglobin concentration [14]. Thus, based on these characteristics and advantages, PA imaging is a powerful tool in stroke research.

Since the focal point of our PA probe is 10 mm, the probe must be very close to the sample. For a probe without charge-coupled device (CCD) guidance, precise positioning is difficult to achieve. Therefore, we combined the CCD camera with a photoacoustic platform to achieve precise positioning in animal experiments. Because the cranial window of the rat is only approximately 6 × 8 mm, the field of view is small and blocked by the PA probe and corresponding optical components, increasing the difficulty of accurate positioning when the skull is removed from the rat. In previous positioning methods, a metal needle is placed on a water tank, and the PA probe is used to scan the position of the metal needle. The strongest ultrasonic signal of the metal needle indicates the target location; then, the metal needle is removed. However, this system obtains inaccurate positioning, possibly resulting in errors that may offset the scan position. Thus, to solve this positioning problem, we combined a PA system with a CCD camera and used the CCD camera to obtain real-time images to identify the position of the PA system B-scan and achieve precise positioning. To assess the imaging capabilities of the designed visible CCD camera-guided photoacoustic imaging (ViCPAI) system and the positioning capabilities of the CCD camera, in vitro carbon fiber and in vivo animal experiments were carried out. For the ViCPAI system, precise and reproducible positioning are both critical. The position of the bregma is difficult to determine after the skull is removed, resulting in inaccurate positioning. Therefore, before the skull is removed, we use the bregma as a base point and introduce two marks in the primary somatosensory cortex of the forelimb (S1FL) area (A-P: +1 mm; M-L: ±4 mm); then, we apply the ViCPAI system to locate the target site and record the scan coordinates. After the skull is removed, the automated three-axis system is used to move the PA probe to the prerecorded coordinates, thereby achieving positioning and reproducible positioning.

Moreover, we used photochemical methods to induce photothrombotic ischemic (PTI) stroke. In addition to affecting brain functions, stroke also changes the behavior of rats. To observe changes after PTI stroke, the blood vessels in the S1FL region in the right hemisphere were investigated. Since the right S1FL region responds to left forelimb stimulation, the left forelimb of the rat was electrically stimulated, and changes in the cerebral blood volume (CBV) and oxygen saturation (SO_2_) in the S1FL region were monitored before and after PTI stroke. Because precise positioning is needed in the brain, we used a CCD camera to locate the S1FL area. The PA probe was guided to a specific location, and the changes in the CBV and SO_2_ in the bilateral cortex after left forelimb electrical stimulation were recorded. Then, the CCD camera was used to guide the PTI to specific blood vessels, and the changes in the CBV and SO_2_ in the bilateral cortex before and after the stroke were observed. Ischemic stroke causes depolarizing waves, similar to cortical spreading depression (CSD), which is called peri-infarct depolarization (PID), resulting in secondary damage to the brain [23]. To study this problem, we used photoacoustic imaging to observe the CSD induced by KCl. In addition, the CBV and SO_2_ were investigated to understand CSD progression, which is a novel tool for studying peri-infarct depolarization. Finally, we verified that the developed ViCPAI system could be applied to observe CSD in brain cortical regions and monitor PID progression after stroke. Thus, our photoacoustic imaging system is a novel and powerful tool for studying stroke and PID.

## 2. Materials and Methods

### 2.1. Fiber Bundle-Based Illumination Dual-Modality Real-Time Visible CCD Camera-Guided Photoacoustic Imaging System

**Figure 1:**
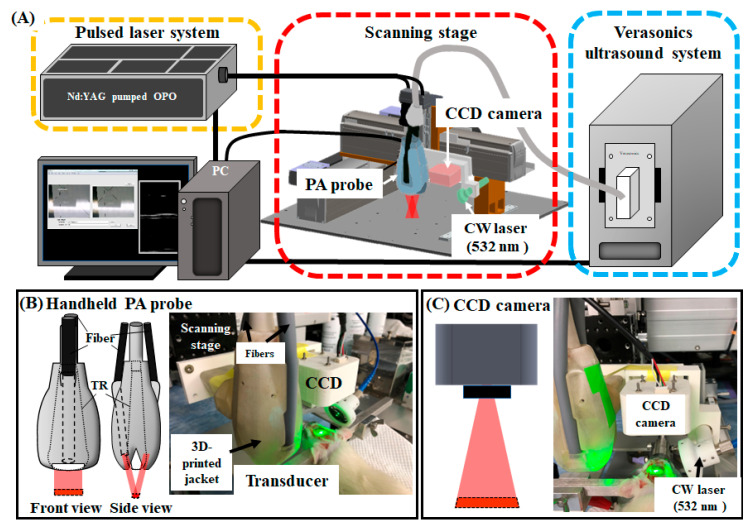
Schematic diagram of the developed dual-modality ViCPAI system with a laser-induced stroke system. (**A**) Illustration of the proposed ViCPAI system. The laser light source was a Nd:YAG laser with an integrated tunable OPO with light wavelengths ranging from 680 to 2400 nm. The OPO produced approximately 7 ns pulses at a 20 Hz repetition rate. The system included an 18.5 MHz high-frequency planar US probe with 128 channels. The probe received PA signals and transmitted and received US signals. (**B**) The system included an 18.5 MHz high-frequency planar ultrasonic probe with a 128-channel US platform for data acquisition. The US transducer had 1286 mm active elements and a −6 dB fractional bandwidth of 67%. The system could receive PA signals and transmit and receive US signals. The components were fixed on the same in-house scanning stage using a 3D-printed holder. (**C**) The PA probe was combined with a CCD camera, and a Sony IMX219 8-megapixel sensor was used as a positioning tool to obtain real-time images. The sensor was 3.674 × 2.760 mm (1/4″ format) and had a 3280 × 2464-pixel resolution for static images. The CCD camera was mounted inside the 3D-printed carrier, and a 3D-printed holder was used to combine the US transducer with the CCD camera carrier. The CCD camera obtained real-time images and aided in precisely positioning the US transducer in the desired ROI. Then, the CW laser-induced PTI stroke and neurovascular activity were observed. (Abbreviations: US, ultrasound; PA, photoacoustic; Nd:YAG, neodymium-doped yttrium aluminum garnet; OPO, optical parametric oscillator; TR, transducer).

Figure 1A shows the developed ViCPAI system. The Nd:YAG laser system used in the PA system incorporates an adjustable optical parametric oscillator (OPO; SpitLight 600, InnoLas Laser GmbH, Krailling, Germany) that produces light wavelengths ranging from 680 nm to 2400 nm. The OPO generates approximately 7 ns pulses at a 20 Hz repetition rate. The system included an 18.5 MHz high-frequency planar ultrasonic probe (L22-14v, Verasonics Inc., Washington, DC, USA) with a 128-channel US platform (Vantage 128, Verasonics Inc., Washington, DC, USA) that received PA signals and transmitted and received US signals. The US transducer has 1286 mm active elements, and a −6 dB fractional bandwidth of 67%. This probe was combined with a CCD camera and a Sony IMX219 8-megapixel sensor (Raspberry Pi camera V2, Raspberry Pi Foundation, Cambridge, UK). The sensor was 3.674 × 2.760 mm (1/4″ format) and capable of producing 3280 × 2464-pixel static images. The CCD camera was mounted inside a 3D printed base, and a 3D printed holder was used to combine the US transducer and CCD camera, as shown in Figure 1. The CCD camera is used to obtain images and to precisely position the US transducer in the desired region of interest (ROI).

The entire PA/US system was implemented using custom-developed MATLAB software (MATLAB R2020a, MathWorks Inc., Natick, MA, USA). The movement of the scanning position was controlled using an in-house scanning stage. Piezoelectric motors (LMR Liner Motor Rob, Toyo Automation Co., Ltd., Tainan, Taiwan) drove the x- and y-axes, and the z-axis was motorized and adjusted by a three-axis platform (Sigma-koki Co., Ltd., Tokyo, Japan). The acoustic waves received by the PA probe were processed at a frame rate of 20 frames per second to reconstruct images on a computer screen. In PA imaging mode, the energy density of the laser light in the sample surface was approximately 12~14 mJ/cm^2^, which is considerably less than the safety limit (20 mJ/cm^2^) determined by the American National Standards Institute (ANSI) [14].

A photograph of the developed PA probe and CCD camera system is shown in Figure 1B. The two components were fixed on the same in-house scanning stage by a 3D-printed holder [13,21]. The PA probe fiber bundles had rectangular output ends and metal circular input ends [24]. The acrylic water tank’s bottom had a rectangular cutout that was used as an acoustic window to connect the PA probe to the sample. To ensure that water did not leak from the cutout, the cutout was covered with a polyethylene film. A layer of ultrasonic (US) gel was placed between the surface of the specimen and the polyethylene film when the PA probe was submerged in the water tank. The axial and lateral resolutions of the developed PA system were measured by scanning a carbon fiber at a depth of 10 mm and finding the full width at half maximum (FWHM). Our ViCPAI system had the axial and lateral resolutions of 124 and 213 μm, respectively [21].

### 2.2. Preparation of the Craniotomy Animal Model

All procedures were performed in accordance with the Institutional Animal Care and Use Committee (IACUC) of the National Health Research Institute (NHRI), Taiwan (IACUC protocol number: NHRI-IACUC-107100-A, NHRI-IACUC-111023-A). All animals were maintained in a 12 h light/dark cycle at a constant temperature and humidity, and all animals had unrestricted access to food and water. To compare the hemodynamic effects and SO_2_ changes in the bilateral cortex of the S1FL area before and after PTI stroke induced by electrical stimulation, 21 male adult Sprague Dawley (SD) rats (BioLASCO Taiwan Co., Ltd., Taipei, Taiwan) (total *n* = 21) weighing between 250 and 350 g were used in three experiments: an electrical stimulation and PTI stroke experiment (*n* = 5), a 2,3,5-triphenyl tetrazolium chloride (TTC) experiment (control group *n* = 5, PTI group *n* = 5), and a KCl-induced CSD experiment (saline group *n* = 3, KCl group *n* = 3). The rats were anesthetized with 1–3% isoflurane (Panion & BF Biotech Inc., Taipei, Taiwan) in oxygen and secured on a stereotaxic frame (Stoelting Co., Wood Dale, IL, USA). Then, each skull was exposed through an incision in the skin. A high-speed drill was applied to expose an approximately 6 mm anterior-posterior (A-P) by 8 mm medial-lateral (M-L) cranial window centered at the bregma to keep the dura intact for craniotomy.

### 2.3. Electrical Stimulation and PTI Stroke Protocols for PA Imaging

**Figure 2:**
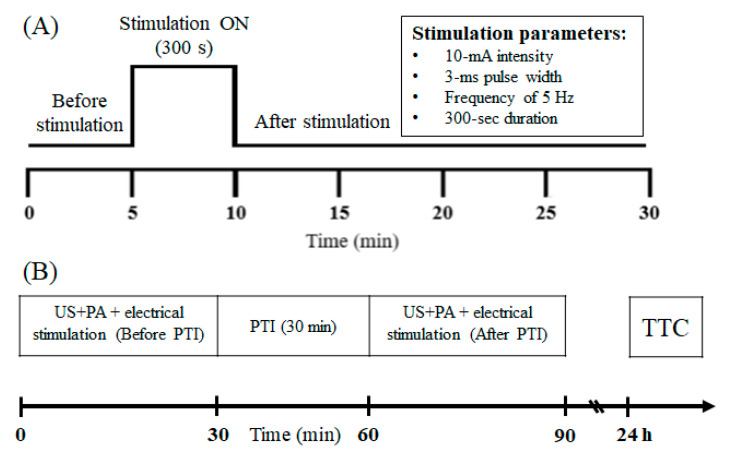
Protocols for electrical left forelimb stimulation and US/PA scans of selected blood vessel and S1FL regions. (**A**) PA signals were collected for 30 min. The first 300 s was used to determine the baseline level. Then, a constant 10 mA electrical stimulation with a pulse width of 3 ms was provided at a frequency of 5 Hz for 300 s. (**B**) Experimental protocol timeline for PTI and electrical stimulation experiments. Scanning US and PA images were obtained in the S1FL region of rats before PTI stroke, and electrical stimulation was applied to observe CBV and SO_2_ changes in the S1FL region. The baseline was collected for 5 min, followed by electrical stimulation for 5 min, and signals were collected for 20 min after the end of the electrical stimulation. Thirty minutes after PTI-induced stroke, the ViCPAI system was used to collect PA signals in the S1FL region to observe the changes induced by electrical stimulation. After 24 h, TTC was used to observe the infarct size after PTI stroke.

Electrical stimulation and PTI stroke were considered in the PA imaging experiment (total *n* = 5). For PA electrical stimulation data collection, somatosensory evoked potential (SSEP) was induced via a stimulator (Isolated pulse stimulator model 2100, A-M Systems Inc., Sequim, WA, USA) to generate a monophasic constant current with a 3 ms pulse width, a 10 mA intensity, and a 5 Hz frequency during the 5 min stimulation period. The needle electrodes were implanted into the rat’s left forelimb, and peripheral sensory electrical stimulation was administered to elicit neurovascular responses in the ischemic cortical region of the primary somatosensory cortex (S1FL). The stimulation was applied for 5 min. A schematic of the PA imaging system is shown in Figure 2A. Briefly, PA signals were recorded for 30 min, with the 5 min before electrical stimulation was applied serving as a baseline for calculations. As shown in Figure 2B, PA signals were recorded before and after PTI stroke. The PTI-induced stroke persisted for 30 min, which was followed by TTC staining 24 h after PTI stroke [25,26].

For PTI stroke PA data collection, the vascular response (changes in SO_2_) before and after ischemic stroke induction was compared to determine whether neural function was impaired or improved after treatment. The hemodynamic changes were identified by observing changes in the PA_800_ signal. In addition, the PA system was applied to assess neurovascular responses to peripheral sensory stimulation after stroke induction.

### 2.4. The Positioning of the ViCPAI System In Vitro and In Vivo

**Figure 3:**
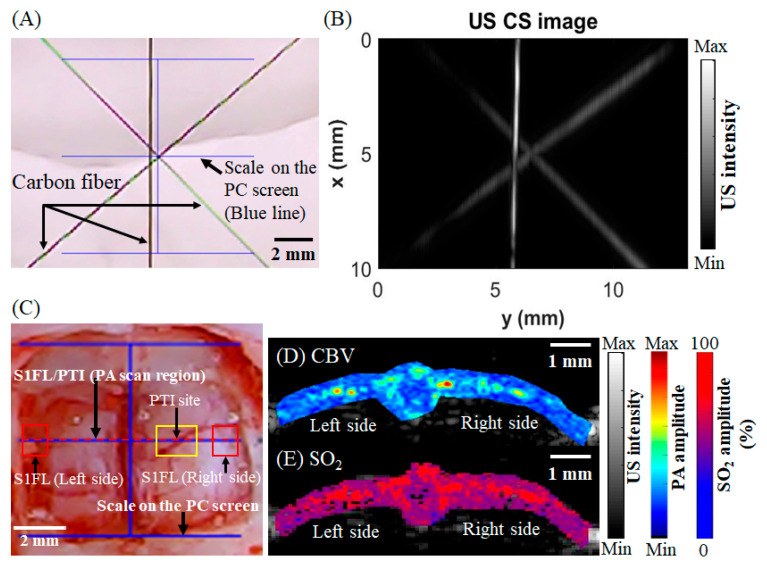
The positioning capability of the CCD camera was used to obtain the PA/US signals of rats after craniotomy and analyze the CBV and SO_2_ values. (**A**) CCD camera view showing a photograph of a carbon fiber sample. The blue lines serve as scale bars and position markers to aid in positioning the US transducer. The four blue lines represent 10 mm in length. (**B**) Three-dimensional reconstruction of a US carbon fiber image (scale bar: 2 mm). (**C**) Positioning of the CCD camera on the brain of a rat after craniotomy. The scan area of +1 mm in the bregma is shown with the red dotted line. The yellow frame indicates the PTI site for the PTI-induced stroke, and the red frame denotes the S1FL region (scale bar: 2 mm). (**D**) The US/PA CBV results of a rat after craniotomy. The scan region is indicated by the red dotted line (scale bar: 1 mm). (**E**) The US/PA or SO_2_ results of a rat after craniotomy (scale bar: 1 mm). (Abbreviations: US, ultrasound; PA, photoacoustic; S1FL, primary somatosensory cortex of the forelimb; PTI, photothrombotic ischemia; CBV, cerebral blood volume; CS, C-scan).

First, we calibrated the positions of the ultrasonic probe and CCD camera to determine their relative coordinates before positioning. We prepared the water tank, placed a metal needle that was sensitive to ultrasound in the tank, and directed the CCD camera at the metal needle. The images obtained by the CCD camera were sent to a computer in real time. We used MATLAB to display a blue line in the center of the CCD camera screen, as shown in Figure 3A. We aligned the center point of the “王” symbol in the CCD image with the metal needle, which was placed horizontally in the water tank, and used the automated three-axis system to set the coordinates of this position as X = 0 μm and Y = 0 μm. Then, the ultrasonic probe was moved to the position of the metal needle, and ultrasonic probe signals were obtained in real time. The signal strength was used as a judgment basis, and the three-axis system was used to adjust the probe to the center of the screen and the position where the signal was the largest. Then, the three-axis system was used to record the current coordinates of the ultrasonic probe. The coordinates were X = −65,000 μm and Y = 9300 μm. Therefore, we can obtain the origin coordinates based on the CCD camera results and the relative coordinates based on the ultrasonic probe results. As long as the device is not moved, the system does not need to be recalibrated.

In the in vitro experiment, after the carbon fiber was arranged, we used a CCD camera to align the center point of the “王” symbol on the screen with the carbon fiber, as shown in Figure 3A. Then, we input the relative coordinates of the calibrated probe into the three-axis system, controlled the probe to move to the appropriate coordinates, and activated the ultrasonic probe to collect carbon fiber signals at this position, as shown in Figure 3B. In the in vivo experiment, we used a CCD camera to locate the S1FL area in a rat and aligned the line in the center of the “王” symbol on the screen with the skull on both sides of the rat to mark the S1FL area in advance, as shown in Figure 3C. We set these coordinates to X = 0 μm and Y = 0 μm, input the relative coordinates of the calibrated probe into the three-axis system, controlled the probe to move to the appropriate coordinates, and used multiple wavelengths (750 nm, 800 nm, and 850 nm) to scan the rat brain. The CBV was determined with the 800 nm wavelength, as shown in Figure 3D. The SO_2_ concentration was determined with the 750 nm and 850 nm wavelengths; the oxyhemoglobin (HbO) and deoxyhemoglobin (Hb) concentrations were converted to obtain the blood oxygen saturation concentration, as shown in Figure 3E. The detailed conversion formula is introduced later.

### 2.5. Determining Blood Oxygen Saturation According to the PA Signals

PA data were acquired at the absorption wavelengths dominated by deoxyhemoglobin (λ = 750 nm) and oxyhemoglobin (λ = 850 nm). The hemoglobin oxygen saturation (SO_2_) was calculated as follows:

(1) $$\mu_{a}^{\lambda }=\varepsilon_{\mathrm{HbO}}^{\lambda }\left[\mathrm{HbO}\right]+\varepsilon_{\mathrm{Hb}}^{\lambda }\left[\mathrm{Hb}\right]$$

where [Hb] and [HbO] are the concentrations of deoxyhemoglobin and oxyhemoglobin, respectively; $\varepsilon_{\mathrm{Hb}\;}^{\lambda }$ and $\varepsilon_{\mathrm{HbO}}^{\lambda }$ are the molar extinction coefficients; and $\mu_{a}^{\lambda }$ is the PA signal [27,28]. The following equation relates the optical absorption coefficient to the acoustic pressure:

(2) $$\mu_{a}^{\lambda }=\frac{P}{\Gamma F}$$

where P is the acoustic pressure, Γ is the Grüneisen coefficient, and F is the optical fluence. Then, the following equation can be used to determine SO_2_ according to [Hb] and [HbO] [8,21,22,24]:

(3) $${\mathrm{SO}}_{2}=\frac{\left[\mathrm{HbO}\right]}{\left[\mathrm{HbO}\right]+\left[\mathrm{Hb}\right]}$$

### 2.6. An Animal Stroke Model of Photothrombotic Ischemia (PTI) for Focal Ischemic Stroke Induction

Cerebral arterioles in a distal branch of the middle cerebral artery (MCA) in the right S1FL area were targeted in a focal ischemic stroke model using a photothrombotic ischemia technique [22]. The photosensitizer Rose Bengal (Sigma Aldrich, St. Louis, MO, USA) was prepared at 10 mg/mL in saline and infused via tail vein injection [29]. The cerebral arteriole was chosen for obstruction using a 5 mW continuous wave (CW) laser at 532 nm and was illuminated for 30 min until a stabilized thrombus formed [30].

### 2.7. KCl-Induced CSD

The KCl-induced CSD experiment included two groups (total *n* = 6): a saline group (*n* = 3) and the KCl group (*n* = 3). A 6 mm anterior-posterior (A-P) × 8 mm medial-lateral (M-L) window centered on the bregma was made in the rat cranial window, and a hole was created in the skull (A-P = −5.0 mm, M-L = +3.0 mm) to apply 0.9% saline (Taiwan Biotech Co., Ltd., Taoyuan, Taiwan) or 4 M KCl (Potassium Chloride, Crystal, Avantor Inc., Radnor, PA, USA) dissolved in ddH_2_O [31]. Before the start of the experiment, we embedded cotton and PE tubes in the hole and connected a 26 G needle with a syringe containing saline or 4 M KCl to the other end of the PE tube. First, to ensure that the ultrasonic probe did not scan outside the desired range, the CCD camera-guided PA system was used for precise positioning, and the baseline was scanned with a wavelength of 800 nm for 5 min. Then, saline or 4 M KCl was slowly injected into the hole, and the region was scanned with the same 800 nm wavelength for 40 min.

### 2.8. TTC Staining

TTC (2,3,5-triphenyl tetrazolium chloride; T8877-25G, Sigma Aldrich, St. Louis, MO, USA) staining was used to quantify the brain infarction. In addition, we used a TTC control group (*n* = 5) and compared the infarct changes with those in the PTI stroke group (*n* = 5). Twenty-four hours after effective PTI introduction, the brains were sliced and incubated with 2% TTC for 20 min in the dark [32]. ImageJ software (v.1.53, National Institutes of Health, Bethesda, MD, USA) was used to evaluate the size of the ischemic infarction and the integrated volume. When stained with TTC, healthy tissue becomes red, while damaged tissue remains white.

### 2.9. Experimental Data Quantitative Analysis Method

In the PA electrical stimulation experiments, hemodynamic changes were determined by observing changes in the PA_800_ signals using an 800 nm wavelength laser. PA data were acquired at the absorption wavelengths dominated by deoxyhemoglobin (λ = 750 nm) and oxyhemoglobin (λ = 850 nm). By calculating the changes in the PA signals with wavelengths of 750 nm and 850 nm, the relative blood oxygen saturation information can be obtained. In the equation $\mu_{a}^{\lambda }=\varepsilon_{\mathrm{HbO}}^{\lambda }\left[\mathrm{HbO}\right]+\varepsilon_{\mathrm{Hb}}^{\lambda }\left[\mathrm{Hb}\right]$, the [HbO] and [Hb] concentrations can be obtained by substituting the photoacoustic signal ($\mu_{a}^{\lambda }$) and molar extinction coefficients ($\varepsilon_{\mathrm{HbO}}^{\lambda }{\;\mathrm{and}\;\varepsilon }_{\mathrm{Hb}}^{\lambda })$ into the formula and then using the ${\mathrm{SO}}_{2}=\frac{\left[\mathrm{HbO}\right]}{\left[\mathrm{HbO}\right]+\left[\mathrm{Hb}\right]}$ formula, allowing us to determine the SO_2_ value.

Since the laser in the PA system is affected by the temperature and humidity of the environment, it is quite difficult to keep it constant. Therefore, we added a laser energy sampling mirror to the laser system, sent the monitored energy to the PC for energy monitoring and sampling, and included the change in the laser energy in the formula for SO_2_ to reduce the influence of the energy change on SO_2_. The PA energy compensation formula is as follows [33]:

(4) $$\mu_{a}^{\lambda }\left(\lambda 750\right)=\frac{P}{{\Gamma F}_{750}}=F_{750}\cdot 〔\varepsilon_{\mathrm{HbO}}^{\lambda }\left[\mathrm{HbO}\right]+\varepsilon_{\mathrm{Hb}}^{\lambda }\left[\mathrm{Hb}\right]〕$$

(5) $$\mu_{a}^{\lambda }\left(\lambda 850\right)=\frac{P}{{\Gamma F}_{850}}=F_{850}\cdot 〔\varepsilon_{\mathrm{HbO}}^{\lambda }\left[\mathrm{HbO}\right]+\varepsilon_{\mathrm{Hb}}^{\lambda }\left[\mathrm{Hb}\right]〕$$

where F_750_ is the laser energy intensity of λ750 and F_850_ is the laser energy intensity of λ850. A more accurate SO_2_ value could be obtained after a substitution into the formula. The [HbO] and [Hb] concentrations could be obtained by substituting the PA signal ($\mu_{a}^{\lambda }$) and the molar extinction coefficients ($\varepsilon_{\mathrm{HbO}}^{\lambda }{\;\mathrm{and}\;\varepsilon }_{\mathrm{Hb}}^{\lambda })$ into the formula and then using the ${\mathrm{SO}}_{2}=\frac{\left[\mathrm{HbO}\right]}{\left[\mathrm{HbO}\right]+\left[\mathrm{Hb}\right]}$ formula, allowing us to determine the SO_2_ value after energy compensation. Then, the scan results in the S1FL region allowed us to determine the average PA_800_ intensity and a relative blood oxygen saturation in this region. The largest S1FL area was approximately A-P + 1.0 mm and M-L + 3.0 to +5.0 mm in the bregma [14]. Then, PTI stroke was induced, and the changes in PA_800_ (i.e., CBV) intensity and relative blood oxygen saturation over time before and after PTI stroke were observed. The CBV signal and SO_2_ values were analyzed each minute. Then, the CBV signal and SO_2_ values at each minute were averaged during the baseline, electrical stimulation, and post-electrical stimulation measurements. Moreover, one-way ANOVA was used in statistical analyses, and a *p*-value < 0.05 was regarded as statistically significant in this work.

We identified changes in the CBV and SO_2_ values before and after PTI stroke in specific blood vessels. Then, the ROI in the S1FL region was circled, and quantitative analyses were performed. In the quantitative analyses, we obtained images and data from five rats before and after stroke. The data were used in statistical analyses, and *p* < 0.05 represents a significant difference in the *t*-tests.

## 3. Results

### 3.1. Obtained Significant Changes in CBV and SO_2_ after PTI Stroke through the Developed US/PA Imaging System

**Figure 4:**
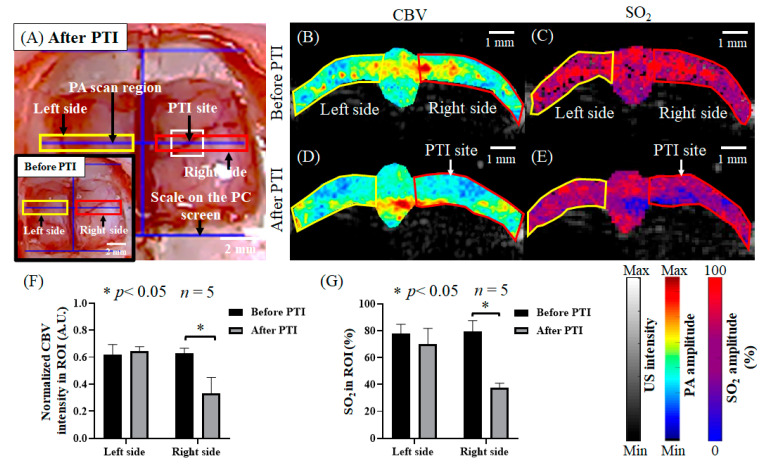
The CBV and SO_2_ values changed before and after PTI strokes in the selected blood vessels. (**A**) White-light images of the rat cranial window before and after stroke. The white frame indicates the laser-induced site for PTI stroke. The yellow frame indicates the scan area in the left hemisphere of the rat (including the S1FL area), and the red frame indicates the scan area in the right hemisphere of the rat (including the S1FL area). (**B**) Photoacoustic imaging of CBV before stroke. The white frame indicates the laser-induced site for PTI stroke. The yellow frame denotes the scan area in the left hemisphere of the rat (including the S1FL area), and the red frame represents the scan area in the right hemisphere of the rat (including the S1FL area). (**C**) Photoacoustic imaging of SO_2_ before stroke. The white frame denotes the laser-induced site for PTI stroke. The yellow frame is the scan area in the left hemisphere of the rat (including the S1FL area), and the red frame is the scan area in the right hemisphere of the rat (including the S1FL area). (**D**) Photoacoustic imaging of CBV after stroke. The white frame is the laser-induced site for PTI stroke. The yellow frame is the scan area in the left hemisphere of the rat (including the S1FL area), and the red frame is the scan area in the right hemisphere of the rat (including the S1FL area). (**E**) Photoacoustic imaging of SO_2_ after stroke. The white frame is the laser-induced site for PTI stroke. The yellow frame is the scan area in the left hemisphere of the rat (including the S1FL area), and the red frame is the scan area in the right hemisphere of the rat (including the S1FL area). (**F**) Quantifying CBV photoacoustic imaging signals before and after stroke. The CBV in the left hemisphere showed no significant difference after stroke. In the right hemisphere, the CBV was 0.44 ± 0.02 before PTI stroke and significantly decreased to 0.26 ± 0.13 after PTI stroke. A significant difference (*p* < 0.05, *t*-test) is shown by the symbol “*”. The data are shown as the mean ± SEM, and there are 5 members in each group. (**G**) Quantifying SO_2_ photoacoustic imaging signals before and after stroke. The SO_2_ levels in the left hemisphere were not significantly different after stroke. In the right hemisphere, SO_2_ was 70% ± 6.9% before PTI stroke and significantly decreased to 37% ± 2.0% after PTI stroke. A significant difference (*p* < 0.05, *t*-test) is shown by the symbol “*”. The data are shown as the mean ± SEM, and there are 5 members in each group.

First, to test the application of the developed PA system in stroke research, we induced PTI stroke in a particular blood vessel and observed the changes in the CBV and SO_2_. After PTI stroke in the right brain, the CBV (*p* < 0.05) and SO_2_ (*p* < 0.05) in the right brain area showed significant downward trends. The changes before and after stroke were clearly obtained; thus, our proposed system can be used as a powerful tool for studying stroke. Figure 4A shows white-light images of the rat cranial window before and after stroke. Figure 4B presents photoacoustic images of the CBV before stroke. Figure 4C shows photoacoustic images of the SO_2_ concentration before stroke. Figure 4D provides photoacoustic images of the CBV after stroke. Figure 4E shows photoacoustic images of the SO_2_ concentration after stroke. Then, photoacoustic imaging signals of the CBV before and after stroke were quantified. The left hemisphere CBV was not significantly different after stroke. However, in the right hemisphere, the CBV was 0.44 ± 0.02 before PTI stroke, and after PTI stroke, the CBV was significantly decreased to 0.26 ± 0.13, as shown in Figure 4F. A significant difference (*p* < 0.05, *t*-test) is shown by the symbol “*”. The data are shown as the mean ± SEM, and each group included five members. Moreover, the SO_2_ photoacoustic imaging signals were quantified before and after stroke. The SO_2_ levels in the left hemisphere were not significantly different after stroke. However, in the right hemisphere, SO_2_ was 70% ± 6.9% before PTI stroke, and after PTI stroke, the SO_2_ concentration was significantly decreased to 37% ± 2.0%, as shown in Figure 4G. A significant difference (*p* < 0.05, *t*-test) is shown by the symbol “*”. The data are shown as the mean ± SEM, and each group included five members.

### 3.2. The Bilateral Cortex Changed in Response to Electrical Stimulation before Stroke

**Figure 5:**
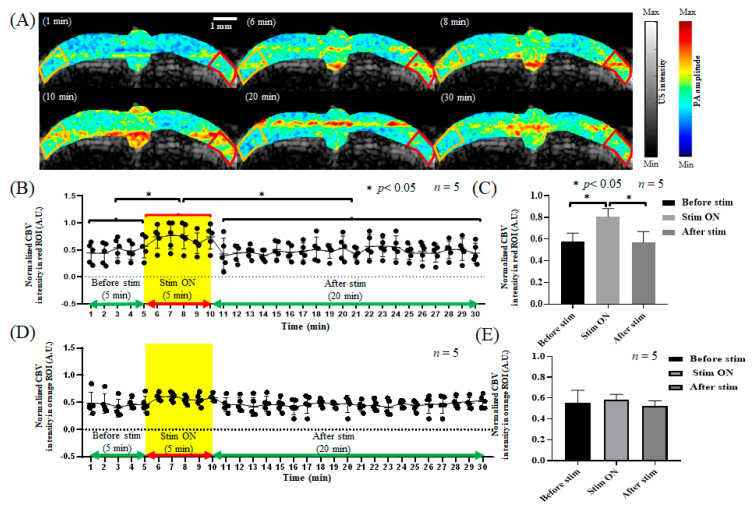
The CBV response in the bilateral cortex to electrical stimulation before stroke. (**A**) The CBV response in the bilateral cortex during 1, 6, 8, 10, 20, and 30 min of electrical stimulation. The left hemisphere of the rat is represented by the orange frame, and the right hemisphere is represented by the red frame, both of which show the S1FL region. (**B**) The CBV trend diagram in the right S1FL region in response to left forelimb electrical stimulation. The yellow frame indicates electrical stimulation. (**C**) The quantification of CBV in the right S1FL region in response to electrical stimulation of the left forelimb. The baseline was 0.56 ± 0.04 before electrical stimulation. The CBV in the right S1FL region increased significantly to 0.78 ± 0.01 during electrical stimulation. After electrical stimulation, the CBV decreased significantly to 0.58 ± 0.01, which is similar to the baseline. A significant difference (*p* < 0.05, *t*-test) is shown by the symbol “*”. The data are shown as the mean ± SEM, and there are 5 members in each group. (**D**) The CBV trend diagram in the left S1FL region in response to electrical stimulation of the left forelimb. The yellow frame indicates electrical stimulation. (**E**) The quantification of CBV in the left S1FL region in response to electrical stimulation of the left forelimb. The CBV in the left S1FL region showed no significant changes in response to electrical stimulation, with values of 0.50 ± 0.01, 0.52 ± 0.01, and 0.49 ± 0.01 before, during, and after electrical stimulation, respectively. The data are shown as the mean ± SEM, and there are 5 members in each group.

**Figure 6:**
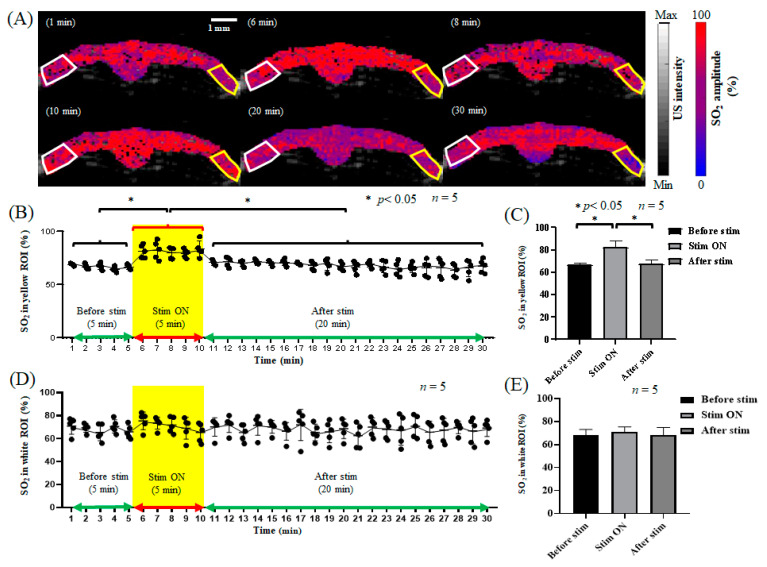
The SO_2_ response in the bilateral cortex to electrical stimulation before stroke. (**A**) The SO_2_ response in the bilateral cortex during 1, 6, 8, 10, 20, and 30 min of electrical stimulation. The left hemisphere of the rat is represented by the white frame, and the right hemisphere is represented by the yellow frame, both of which show the S1FL region. (**B**) The SO_2_ trend diagram in the right S1FL region in response to electrical stimulation of the left forelimb. The yellow frame indicates electrical stimulation. (**C**) The quantification of SO_2_ in the right S1FL region in response to electrical stimulation of the left forelimb. The baseline was 64% ± 0.1% before electrical stimulation. The SO_2_ level in the right S1FL region increased significantly to 82% ± 2.4% during electrical stimulation. After electrical stimulation, the SO_2_ level decreased significantly to 65% ± 0.1%, which is similar to the baseline. A significant difference (*p* < 0.05, *t*-test) is shown by the symbol “*”. The data are shown as the mean ± SEM, and there are 5 members in each group. (**D**) The SO_2_ trend in the left S1FL region in response to electrical stimulation of the left forelimb. The yellow frame indicates electrical stimulation. (**E**) The quantification of SO_2_ in the left S1FL region in response to electrical stimulation of the left forelimb. The SO_2_ in the left S1FL region showed no significant changes in response to electrical stimulation, with values of 63% ± 2%, 65% ± 1%, and 62% ± 3% before, during, and after electrical stimulation, respectively. The data are shown as the mean ± SEM, and there are 5 members in each group.

After electrical stimulation of the left forelimb and before stroke, the CBV and relative SO_2_ in the S1FL area of the right brain increased due to electrical stimulation, as shown in Figure 5A and Figure 6A. The CBV increased from 0.55 ± 0.085 to 0.79 ± 0.036, and after the end of electrical stimulation, the CBV decreased from 0.79 ± 0.036 to 0.57 ± 0.05, as shown in Figure 5B,C, while the relative SO_2_ level increased from 66.66% ± 0.577% to 85.33% ± 4.041% in response to electrical stimulation and decreased to 66.67% ± 3.786% after electrical stimulation, as shown in Figure 6B,C. The results show that during electrical stimulation of the left forelimb, the CBV and relative SO_2_ in the brain both increased significantly. Moreover, the CBV in the left S1FL region increased from 0.538 ± 0.074 to 0.578 ± 0.072 during electrical stimulation and decreased to 0.518 ± 0.025 after electrical stimulation. Figure 5D,E show that the relative SO_2_ level increased from 66.0% ± 5.196% to 68.66% ± 3.786% during electrical stimulation and decreased to 65.0% ± 6.557% after electrical stimulation. However, as shown in Figure 6D,E, this change was not significant. Thus, the results indicate that the right S1FL area responded to electrical stimulation of the left forelimb, enhancing the changes in neurovascular function, while the left brain was not affected by electrical stimulation of the left forelimb, with no significant changes observed.

### 3.3. The Bilateral Cortex Changed in Response to Electrical Stimulation after Stroke

**Figure 7:**
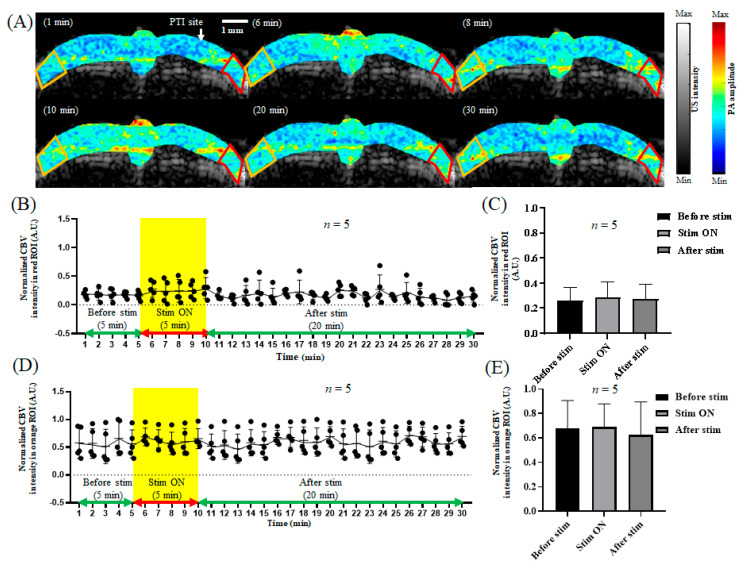
The CBV response in the bilateral cortex in response to electrical stimulation after stroke. (**A**) The CBV response in the bilateral cortex during 1, 6, 8, 10, 20, and 30 min of electrical stimulation. The left hemisphere of the rat is represented by the orange frame, and the right hemisphere is represented by the red frame, both of which show the S1FL region. (**B**) The CBV trend diagram in the right S1FL region in response to electrical stimulation of the left forelimb. The yellow frame indicates electrical stimulation. (**C**) The quantification of CBV in the right S1FL region in response to electrical stimulation of the left forelimb. The CBV in the right S1FL region showed no significant changes in response to electrical stimulation after PTI stroke, with values of 0.35 ± 0.01, 0.39 ± 0.02, and 0.37 ± 0.01 before, during, and after electrical stimulation, respectively. The data are shown as the mean ± SEM, and there are 5 members in each group. (**D**) The CBV trend diagram in the left S1FL region in response to electrical stimulation of the left forelimb. The yellow frame indicates electrical stimulation. (**E**) The quantification of CBV in the left S1FL region in response to electrical stimulation of the left forelimb. The CBV in the left S1FL region showed no significant changes in response to electrical stimulation, with values of 0.52 ± 0.11, 0.58 ± 0.12, and 0.45 ± 0.21 before, during, and after electrical stimulation, respectively. The data are shown as the mean ± SEM, and there are 5 members in each group.

**Figure 8:**
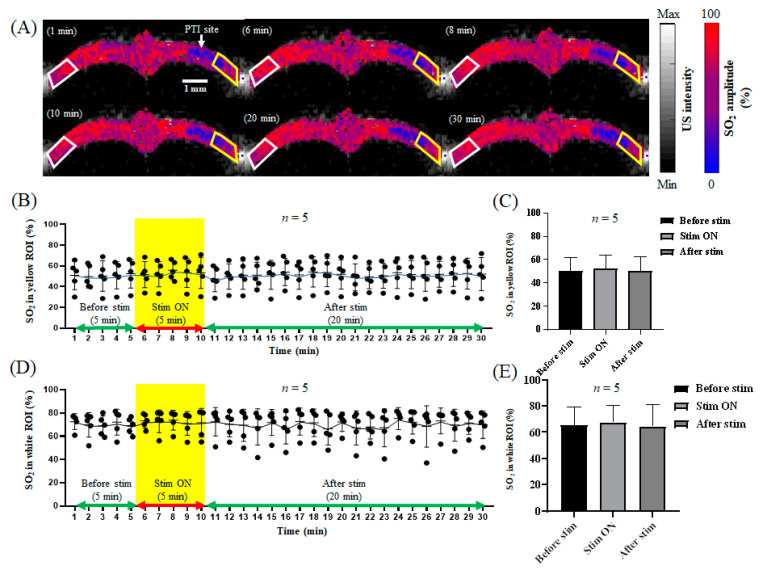
The SO_2_ response in the bilateral cortex to electrical stimulation after stroke. (**A**) The SO_2_ response in the bilateral cortex during 1, 6, 8, 10, 20, and 30 min of electrical stimulation. The left hemisphere of the rat is represented by the white frame, and the right hemisphere is represented by the yellow frame, both of which show the S1FL region. (**B**) The SO_2_ trend diagram in the right S1FL region in response to electrical stimulation of the left forelimb. The yellow frame indicates electrical stimulation. (**C**) The quantification of SO_2_ in the right S1FL region in response to electrical stimulation of the left forelimb. The SO_2_ in the right S1FL region showed no significant changes in response to electrical stimulation after PTI stroke, with values of 42% ± 5.3%, 44% ± 5.1%, and 41% ± 5.8% before, during, and after electrical stimulation, respectively. The data are shown as the mean ± SEM, and there are 5 members in each group. (**D**) The SO_2_ trend diagram in the left S1FL region in response to electrical stimulation of the left forelimb. The yellow frame indicates electrical stimulation. (**E**) The quantification of SO_2_ in the left S1FL region in response to electrical stimulation of the left forelimb. The SO_2_ in the left S1FL region showed no significant changes in response to electrical stimulation, with values of 58% ± 7.3%, 59% ± 7.1%, and 56% ± 8.1% before, during, and after electrical stimulation, respectively. The data are shown as the mean ± SEM, and there are 5 members in each group.

After focal stroke in rats, we monitored the changes in the CBV and relative SO_2_ levels in the S1FL area of the rat bilateral cortex in response to electrical stimulation of the left forelimb, as shown in Figure 7A and Figure 8A. After stroke, the CBV in the right brain increased slightly from 0.337 ± 0.021 to 0.375 ± 0.029 during electrical stimulation, then decreased to 0.36 ± 0.01, as shown in Figure 7B,C. Moreover, the relative SO_2_ level increased slightly from 45.66% ± 11.846% to 48.24% ± 11.183% in response to electrical stimulation, then decreased to 44.95% ± 10.954% after electrical stimulation, as shown in Figure 8B,C. The changes in the CBV and relative SO_2_ levels were not significant. In addition, after stroke, the CBV in the left brain increased slightly from 0.523 ± 0.128 to 0.572 ± 0.135 during electrical stimulation, then decreased to 0.457 ± 0.204, as shown in Figure 7D,E. The relative SO_2_ level increased slightly from 58.0% ± 13.021% to 60.33% ± 12.055% in response to electrical stimulation, then decreased to 55.21% ± 15.395% after electrical stimulation, as shown in Figure 8D,E. The results show that the right S1FL region did not respond to electrical stimulation of the left forelimb after stroke. Thus, focal stroke caused neurovascular damage in the rat brain, with the CBV and relative SO_2_ levels in the left brain before and after stroke and during electrical stimulation remaining essentially constant, with no significant changes.

### 3.4. Quantification of the Infarct Volume after PTI Stroke

**Figure 9:**
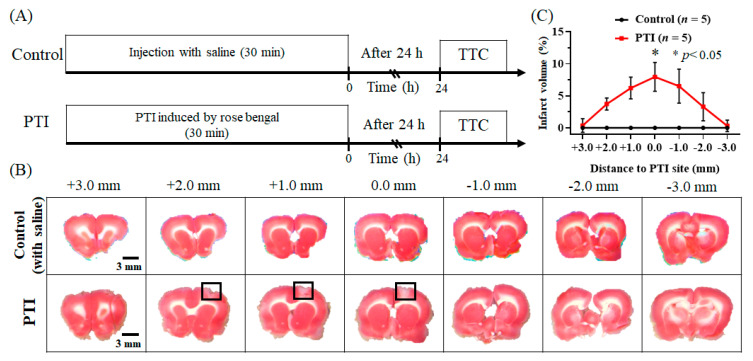
The size of cerebral infarction after stroke was observed by TTC staining. (**A**) The protocol for TTC staining. The control group was subjected to TTC staining 24 h after saline injection. The PTI group was subjected to TTC staining 24 h after injecting rose bengal to induce PTI stroke for 30 min. (**B**) TTC staining showed that the control group had no infarct after sham PTI stroke. Twenty-four hours after PTI stroke, TTC staining showed infarcts with white areas. Infarcts appear within +2 mm and −2 mm of the PTI site, with the black frames indicating infarct areas. (**C**) Compared with the control group (black line), the PTI stroke group (red line) had a significant difference in terms of infarct size. Infarcts were observed within +2 mm and −2 mm of the PTI site, while the PTI core position was 0 mm, and the average infarction area reached up to 6.8% ± 0.335%, which was significantly different than the control group. A significant difference (*p* < 0.05, *t*-test) is shown by the symbol “*”. The data are shown as the mean ± SEM, and there are 5 members in each group.

Twenty-four hours after PTI, brain sections were stained with TTC, and the infarct volume was quantitatively analyzed. An example image of TTC staining is shown in Figure 9B. When stained with TTC, healthy tissue becomes red, while damaged tissue remains white. Compared with the control group, the PTI stroke group had a significant difference in infarct size. The PTI core position was 0 mm, and the average infarction area reached up to 6.8% ± 0.335%, as shown in Figure 9C. The data are shown as the mean ± SEM, and each group included five members.

### 3.5. KCl-Induced CSD Can Be Monitored by the ViCPAI System

**Figure 10:**
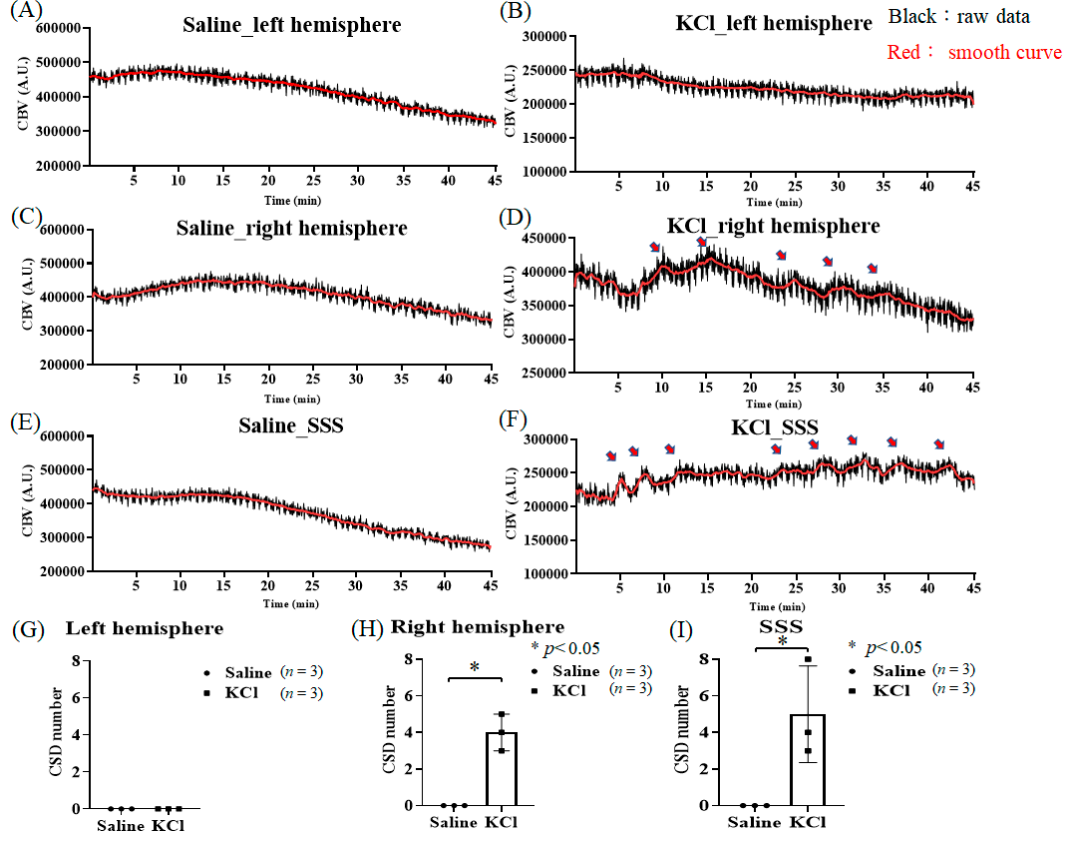
KCl-induced CSD can be monitored by the ViCPAI system. (**A**,**B**) After saline and KCl injection, respectively, into the skull hole in the right hemisphere, no CSD progression was observed in the left hemisphere. (**C**,**D**) After saline and KCl injection, respectively, into the skull hole in the right hemisphere, no CSD progression was observed in the right hemisphere after treatment with saline, while CSD progression was observed in the right hemisphere after KCl treatment (the red arrows represent CSD numbers). (**E**,**F**) After saline and KCl injection, respectively, into the skull hole in the right hemisphere, no CSD progression was observed in the SSS after treatment with saline, while CSD progression was observed in the SSS after KCl treatment (the red arrows represent CSD numbers). (**G**) In the left hemisphere, CSD progression was not observed after saline or KCl injection, and there was no statistical significance. (**H**) According to the statistical results, KCl induced CSD 4 ± 0.577 times on average, with a significant difference compared with the saline group. A significant difference (*p* < 0.05, *t*-test) is shown by the symbol “*”. The data are shown as the mean ± SEM, and there are 3 members in each group. (**I**) According to the statistical results, KCl induced CSD 5 ± 1.528 times on average, with a significant difference compared with the saline group. A significant difference (*p* < 0.05, *t*-test) is shown by the symbol “*”. The data are shown as the mean ± SEM, and there are 3 members in each group.

The ViCPAI system was used to guide the PA probe to the target position, and then saline or KCl was used to observe CSD progression. Figure 10A shows that no CSD was observed in the left hemisphere after saline injection in the right-hemisphere cranial foramen. Figure 10B indicates that no CSD was observed in the left hemisphere after KCl injection into the skull hole in the right hemisphere. Figure 10C demonstrates that as expected, CSD was not observed in either hemisphere, and the changes were not statistically significant. Figure 10D shows that no CSD was observed in the right hemisphere after saline injection into the skull hole in the right hemisphere. Figure 10E shows CSD progression in the right hemisphere after KCl injection into the skull hole in the right hemisphere. According to the statistical results, the KCl injection increased CSD by 4 ± 0.577 times on average, with a significant difference compared with the saline group, as shown in Figure 10F. A significant difference (*p* < 0.05, *t*-test) is shown by the symbol “*”. The data are shown as the mean ± SEM, and each group included five members. Figure 10G shows that no CSD was observed in the right hemisphere after saline injection into the skull hole in the right hemisphere. Figure 10H demonstrates CSD progression in the right hemisphere after KCl injection into the skull hole in the right hemisphere. According to the statistical results, the KCl injection increased CSD by 5 ± 1.528 times on average, with a significant difference compared with the saline group, as shown in Figure 10I. A significant difference (*p* < 0.05, *t*-test) is shown by the symbol “*”. The data are shown as the mean ± SEM, and each group included three members.

## 4. Discussion

### 4.1. Visible CCD Camera-Guided PA/US Imaging System Can Potentially Achieve Precise Positioning

In neuroimaging, CT, fMRI, and PET techniques offer morphological views of the brain with good spatial resolution, enabling multiparametric analyses of brain tissue characteristics in terms of functional and structural information [34]. Therefore, instruments with different functions should be integrated to emphasize the complementarity of different tools [11], such as PET-CT and PET-MRI. These systems can precisely target tissue, allowing researchers to determine appropriate treatments [35]. However, in this study, we required instruments that are suitable for small animal experiments with precise positioning capabilities. The PA system has a high spatial resolution and provides visual imaging information for observing cerebral hemodynamics [36]. However, the existing PA system cannot locate regions, and the position scanned by the PA probe must be determined manually.

The limited positioning capability of the PA system increases the difficulty of scanning specific areas in the rat brain. Since different brain regions in the rat are small and located near one another, if the error exceeds 1 mm, the target location may not be obtained. Because the focal point of the photoacoustic probe is located at 10 mm, the probe must be very close to the sample. For probes without CCD guidance, precise positioning is difficult to achieve. Thus, we combined a CCD camera with the photoacoustic platform to achieve precise positioning in animal experiments. Therefore, an accurate positioning system is needed as an auxiliary to help the system locate the target area quickly and accurately. A 3D-printed carrier was used to connect the CCD camera and PA system, and the CCD camera was mounted on the carrier, as shown in Figure 1B,C. Since the PA system was set up on an automated three-axis system, the PA probe could act synchronously after being combined with the CCD camera without affecting the positioning accuracy. Since the minimum measurement precision required in the rat brain was 0.01 mm and the S1FL area ranged from A-P: +2.52 mm to −1.44 mm, the largest position in the S1FL area was in A-P: +1 mm. Moreover, the minimum step distance of the automated three-axis system was 1 μm, which is sufficient for obtaining precise positioning. In summary, the automated three-axis system is very helpful for achieving precise positioning.

Since the distribution of blood vessels on the surface of each rat differs after craniotomy, the ViCPAI system must achieve not only precise positioning but also reproducible positioning. The position of the bregma is difficult to determine after craniectomy, and the bregma cannot be accurately located. Thus, before the skull is removed, we made two marks (A-P: +1 mm; M-L: ±4 mm) in the S1FL area based on the location of the bregma and then located and recorded the current scan coordinates with the ViCPAI system. After skull removal, the PA probe was moved to the prerecorded coordinates by using the automated three-axis system, thus achieving reproducible positioning.

Then, in in vitro and in vivo experiments, the positioning ability of the ViCPAI system was evaluated using carbon fiber and the rat brain, respectively, as shown in Figure 3. With the guidance of the CCD camera, the PA probe can reach the target location and achieve precise positioning results. In the stroke experiment, the ViCPAI system was used to assess the changes in CBV and SO_2_ in cerebral hemodynamics before and after PTI stroke, as shown in Figure 4. The results showed that the CBV and SO_2_ values decreased significantly after PTI stroke.

### 4.2. Changes in Cerebral Hemodynamics in Response to Left Forelimb Electrical Stimulation before and after PTI Stroke

To understand the functionality of the ViCPAI system, the system was applied in animal experiments. The hemodynamic changes in the S1FL region in the brain in response to electrical stimulation of the rat’s left forelimb were observed. The narrow blood vessels in the S1FL area require precise positioning. Therefore, introducing a CCD camera to the PA system assists in more accurately obtaining data in specific areas in animal experiments.

Before PTI stroke, the hemodynamic changes in the cerebral cortex in response to left forelimb electrical stimulation were investigated, and the PA signals in the right hemisphere significantly increased in response to electrical stimulation (*p* < 0.05). The data are shown as the mean ± SEM, and each group included five members. In contrast, the left side of the brain had no significant changes, as shown in Figure 5. In terms of relative oxygen saturation, the SO_2_ concentration significantly increased only in the right hemisphere during electrical stimulation (*p* < 0.05). The data are shown as the mean ± SEM, and each group included five members. The left side of the brain showed no significant changes, as displayed in Figure 6.

After PTI stroke, the CBV associated with hemodynamics and the SO_2_ concentration relative to oxygen saturation showed no significant differences in the bilateral cerebral cortex in response to left forelimb electrical stimulation, as shown in Figure 7 and Figure 8. Then, 24 h after PTI stroke, TTC staining was performed. The red part indicates viable cells, and the white part indicates dead damaged cells. When the experimental and control groups were compared, clear infarcts were observed after stroke, as shown in Figure 9.

### 4.3. Observation of KCl-Induced CSD Progression with the ViCPAI System

In a previous study, stroke was shown to cause PID [35] that was similar to CSD waves. The main difference between CSD and PID is the method of induction. CSD waves are depolarization waves generated by chemical, mechanical, or electrical stimulation of cortical neurons that cause transmembrane changes in the ion concentrations of neuronal cells [37]. The main reason for this change is that potassium ions are outside cells, and potassium accumulation causes potassium ions to move into neuronal cells, triggering neuronal depolarization and transient increases in cerebral blood flow (CBF), local tissue oxygen tension, and metabolic rate [38]. Ischemic stroke causes PID. The main reason is that the ischemic core loses the oxygen, glucose, and nutrients transmitted by the blood; thus, neurons lack the adenosine triphosphate (ATP) needed to maintain their functions, resulting in a lack of ATP in the sodium–potassium pump, which is required for controlling the balance of cell ions [39], causing potassium ions to accumulate outside the cell, which subsequently leads to depolarization and increased permeability of the cell membrane.

In this study, we used the ViCPAI system to observe PID after PTI stroke. However, due to hardware equipment limitations, it is difficult to use lasers to induce PTI stroke during PA scanning. Therefore, we simulated the progression of PID with the same method as used in the KCl-induced CSD experiments and investigated whether hemodynamic changes could be observed. A hole for injecting saline or KCl was made in the lower right corner of the cerebral cranial window, and hemodynamic changes were observed in the left cerebral cortex, right cerebral cortex, and superior sagittal sinus (SSS) after injection of saline or KCl. As a result, CSD progression could be monitored only in the right cerebral cortex and SSS after KCl infusion. Compared with saline, *p* values < 0.05 were considered significant in the right cerebral cortex and SSS, respectively. The data are shown as the mean ± SEM, and each group included three members, as shown in Figure 10. The results show that the average CSD value in the right cerebral cortex was 4 ± 0.577, and the average CSD value in SSS was 5 ± 1.528. The CSD results are the same as those in a previous study by Amir Ghaemi et al. [40]. According to our results, CSD can be well detected by the ViCPAI system. In addition, ECoG or other optical imaging systems can be used to detect CSD, such as laser speckle contrast imaging (LSCI) and laser Doppler flowmetry (LDF). We demonstrate that the proposed ViCPAI system is an emerging detection tool.

## 5. Conclusions

In this study, we combined a CCD camera and PA probe in an integrated system, allowing the PA probe to achieve precise positioning. The CCD camera was introduced as a positioning tool to accurately deliver the ultrasonic probe to the desired measurement location. The ViCPAI UI interface and automated three-axis system ensure that the PA probe can be moved to the previous position coordinates, thereby achieving reproducible positioning. This function is necessary for animal experiments and can improve positioning when data are lacking. To examine the feasibility of the ViCPAI system, we investigated focal PTI stroke in the right cerebral blood vessel and observed changes in the CBV and SO_2_ of the blood vessels before and after stroke. The results showed significant decreases in both CBV and SO_2_, confirming that our photoacoustic system can be applied in neuroscience research. Then, we used the developed photoacoustic system to observe changes in the S1FL area of the bilateral cortex in response to left forelimb electrical stimulation before and after stroke. Left forelimb electrical stimulation significantly increased the CBV and SO_2_ in the right S1FL region before stroke. However, there was no significant change in the right S1FL region in response to electrical stimulation after stroke, indicating that the function of the S1FL region is severely impaired by stroke. Moreover, the CBV and SO_2_ in the S1FL area of the left brain did not change in response to electrical stimulation before or after stroke. In future work, we will use the ViCPAI system to observe CSD progression induced by 1 M and 4 M KCl and PID progression induced by PTI stroke to confirm whether our photoacoustic system can be used to evaluate CSD and PID. Moreover, in future work, we plan to introduce a laser fiber near the US probe, allowing us to simultaneously use the laser to induce stroke while the US probe is scanning and record the PID progression. In other words, from the baseline measurement to the PID measurements after induced stroke, the probe will not need to move, thereby allowing uninterrupted scanning and ensuring the integrity of the experiment.

## Data Availability

Data will be provided upon request by the corresponding author (Lun-De Liao) of this article.
